# Reply to: When did mammoths go extinct?

**DOI:** 10.1038/s41586-022-05417-2

**Published:** 2022-11-30

**Authors:** Yucheng Wang, Ana Prohaska, Haoran Dong, Adriana Alberti, Inger Greve Alsos, David W. Beilman, Anders A. Bjørk, Jialu Cao, Anna A. Cherezova, Eric Coissac, Bianca De Sanctis, France Denoeud, Christoph Dockter, Richard Durbin, Mary E. Edwards, Neil R. Edwards, Julie Esdale, Grigory B. Fedorov, Antonio Fernandez-Guerra, Duane G. Froese, Galina Gusarova, James Haile, Philip B. Holden, Kristian K. Kjeldsen, Kurt H. Kjær, Thorfinn Sand Korneliussen, Youri Lammers, Nicolaj Krog Larsen, Ruairidh Macleod, Jan Mangerud, Hugh McColl, Marie Kristine Føreid Merkel, Daniel Money, Per Möller, David Nogués-Bravo, Ludovic Orlando, Hannah Lois Owens, Mikkel Winther Pedersen, Fernando Racimo, Carsten Rahbek, Jeffrey T. Rasic, Alexandra Rouillard, Anthony H. Ruter, Birgitte Skadhauge, John Inge Svendsen, Alexei Tikhonov, Lasse Vinner, Patrick Wincker, Yingchun Xing, Yubin Zhang, David J. Meltzer, Eske Willerslev

**Affiliations:** 1 Department of Zoology, University of Cambridge, Cambridge, UK; 2 Lundbeck Foundation GeoGenetics Centre, Globe Institute, University of Copenhagen, Copenhagen, Denmark; 3 ALPHA, State Key Laboratory of Tibetan Plateau Earth System, Environment and Resources (TPESER), Institute of Tibetan Plateau Research (ITPCAS), Chinese Academy of Sciences (CAS), Beijing, China; 4 Key Laboratory of Western China’s Environmental Systems (Ministry of Education), College of Earth and Environmental Science, Lanzhou University, Lanzhou, China; 5 Génomique Métabolique, Genoscope, Institut François Jacob, CEA, CNRS, Univ Evry, Université Paris-Saclay, Evry, France; 6 Institute for Integrative Biology of the Cell (I2BC), Université Paris-Saclay, CEA, CNRS, Gif-sur-Yvette, France; 7 The Arctic University Museum of Norway, UiT—The Arctic University of Norway, Tromsø, Norway; 8 Department of Geography and Environment, University of Hawaii, Honolulu, HI USA; 9 Department of Geosciences and Natural Resource Management, University of Copenhagen, Copenhagen, Denmark; 10 Institute of Earth Sciences, St Petersburg State University, St Petersburg, Russia; 11 Arctic and Antarctic Research Institute, St Petersburg, Russia; 12 Université Grenoble-Alpes, Université Savoie Mont Blanc, CNRS, LECA, Grenoble, France; 13 Department of Genetics, University of Cambridge, Cambridge, UK; 14 Carlsberg Research Laboratory, Copenhagen V, Denmark; 15 School of Geography and Environmental Science, University of Southampton, Southampton, UK; 16 Alaska Quaternary Center, University of Alaska Fairbanks, Fairbanks, AK USA; 17 School of Environment, Earth and Ecosystem Sciences, The Open University, Milton Keynes, UK; 18 Center for the Environmental Management of Military Lands, Colorado State University, Fort Collins, CO USA; 19 Department of Earth and Atmospheric Sciences, University of Alberta, Edmonton, Alberta Canada; 20 Faculty of Biology, St Petersburg State University, St Petersburg, Russia; 21 Department of Glaciology and Climate, Geological Survey of Denmark and Greenland, Copenhagen K, Denmark; 22 Department of Earth Science, University of Bergen, Bergen, Norway; 23 Bjerknes Centre for Climate Research, Bergen, Norway; 24 Department of Geology, Quaternary Sciences, Lund University, Lund, Sweden; 25 Center for Macroecology, Evolution and Climate, Globe Institute, University of Copenhagen, Copenhagen Ø, Denmark; 26 Centre d’Anthropobiologie et de Génomique de Toulouse, Faculté de Médecine Purpane, Université Paul Sabatier, Toulouse, France; 27 Center for Global Mountain Biodiversity, Globe Institute, University of Copenhagen, Copenhagen, Denmark; 28 Gates of the Arctic National Park and Preserve, US National Park Service, Fairbanks, AK USA; 29 Department of Geosciences, UiT—The Arctic University of Norway, Tromsø, Norway; 30 Zoological Institute, Russian academy of sciences, St Petersburg, Russia; 31 Resource and Environmental Research Center, Chinese Academy of Fishery Sciences, Beijing, China; 32 College of Plant Science, Jilin University, Changchun, Jilin China; 33 Department of Anthropology, Southern Methodist University, Dallas, TX USA; 34 Wellcome Trust Sanger Institute, Wellcome Genome Campus, Cambridge, UK; 35 MARUM, University of Bremen, Bremen, Germany

**Keywords:** Palaeoecology, Next-generation sequencing, Ecological genetics, Molecular ecology

replying to J. H. Miller & C. Simpson. *Nature* 10.1038/s41586-022-05416-3 (2022)

Since the inception of ancient environmental DNA (eDNA) research, considerable attention has been paid to the depositional and diagenetic processes of DNA molecules in different sediments and settings^1^. Understanding those processes is critical to determine whether the recovered DNA is of the same age as the deposit in which it is found. It is therefore not unreasonable to ask, as Miller and Simpson have^2^ in response to our recently published eDNA study of 50,000 years of Arctic ecosystem changes^3^, whether remains of long-dead megafauna might have contributed older DNA to younger deposits. They propose that this may account for our finding that mammoths persisted into the Holocene epoch in the continental Arctic.

The basis for Miller and Simpson’s proposal is that mammoth remains could have persisted on the surface of cold Arctic landscapes for millennia after the species’ extinction, and while decomposing, released DNA into younger sediment layers. Their argument assumes that surface skeletal persistence is predominantly temperature-related, based on a correlation between mean annual temperature and the time unburied bones appear to persist. Leaving aside the limited sample size (*n* = 10) on which their correlation is derived, and the fact that not all the dated bones in the model have been on the surface since the animals’ death (for example, the Wrangel Island mammoths were evidently released from permafrost only a few years before their discovery^4^), there can be little doubt that temperature is a factor in bone preservation in the Arctic. However, it is not the sole or even dominant factor. Instead, this is a region where multiple factors work against ubiquitous, millennia-long preservation, including carnivore and scavenger activity, moisture effects, seasonal freezing and thawing, strong ultraviolet radiation, and a range of biogeochemical processes that lead to enzyme digestion and organic matter decomposition^5,6^. Mammoth individuals, being large, would require wide geographic ranges^7^. The expected average density of mammoth fossils per unit area would therefore be extremely low, and so too would the likelihood that these rare remains contributed DNA to our sampling sites. Given that mammoth DNA was found in 23 Holocene samples from 14 different sites (Fig. 1a), these late survivals are highly unlikely to be a result of DNA released from dead remains.

**Fig. 1:**
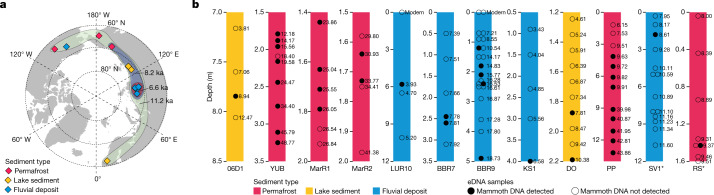
The geographical distribution of late-surviving mammoths, and the vertical distribution of eDNA samples and the identified mammoths in sediment profiles. **a**, Mammoth eDNAs were identified in 23 out of 192 Holocene samples, from 14 out of 32 sites covering the Holocene, and originated from 3 different sediment contexts. The 3 coloured regions show the shrinking distribution of mammoth in the Holocene: green, blue and red correspond to 11.2, 8.2 and 6.6 kyr bp, respectively. **b**, Sites (*n* = 12) where mammoth eDNA was detected in at least one sample and with available sampling depths. For sites with only height available (marked with an ending asterisk in the site name), the sampling heights have been converted to relative depths. The number next to each eDNA sample indicates the age (in kyr bp) of that sample. More details can be found in ref. ^3^.

Furthermore, the eDNA that we obtained from surface samples belonged solely to species present on the landscape presently, indicating that secondary contamination from fossil material is minor. However, it is well understood that some depositional settings (for example, riverbanks and thaw lakes) may be affected by complex processes, whereby older material (not only eDNA but the sediment stratums) can be redeposited within younger sediments. This applied for one site (an actively eroding riverbank setting) of our original study that did not meet our criteria of an unmixed section with clear sedimentological and chronological contexts for eDNA sampling (described in the supplementary information of ref. ^3^), which was therefore excluded from the analysis. This reinforces the well-known caution that fluvial settings require particularly stringent sampling and dating protocols^8^.

Although Miller and Simpson rightly note that there is a near-continuous record of dated mammoth fossils, that record is not a reliable estimator of extinction timing. The youngest dated fossil marks the last time a species was abundant on the landscape^9^, rather than its last occurrence, which is highly likely to go undetected when a species is declining toward extinction, especially across the large geographic range of the vast Arctic landmass. Given the patchy nature of both the fossil and radiocarbon records, there can be centuries-long gaps between dated specimens (figure 1 in ref. ^2^). Those gaps would only increase as species declined and shifted their ranges to smaller portions of their former area^10^. Mammoths may have survived in refugia—such as the last pockets of the steppe-tundra landscape to which they were adapted—long after the date of the last known fossils, and most probably also after their last recorded occurrence in eDNA. However, there is a greater chance of detecting the lingering presence of an animal with eDNA than with its fossils, because an animal releases millions of DNA molecules onto the landscape on a daily basis over the course of its lifetime, but only leaves one skeleton, which is far less likely to be preserved, found and dated.

Notwithstanding limitations in Miller and Simpson’s model and the lack of evidence for redeposition of DNA in our samples, it is reasonable to ask what we might expect to see if the slow decomposition of mammoth tissues on cold Arctic landscapes released DNA into sediments ubiquitously millennia after mammoth extinctions.

First, if redeposition of ancient DNA were widespread, we would expect to see mammoth eDNA in many sampling sites across the species’ full range during the late Pleistocene epoch, and not only restricted to particular regions in the Holocene. Yet, we instead found evidence of later surviving populations—mammoths younger than the Pleistocene/Holocene boundary (11.7 thousand years before present (kyr bp))—in only 23 out of the 192 Holocene samples, in different depositional contexts from 14 out of the 32 sites covering the Holocene (Fig. 1a). The Holocene-age mammoth eDNA occurs in distinct spatial and temporal patterns. It disappears first from the North Atlantic and North American regions, and finally from Siberia, especially northwest and central Siberia (Fig. 1a). These patterns are highly unlikely to have resulted from mammoth bones persisting on the ground surface or being exhumed from below then releasing DNA—if that were the case, the pattern of Holocene ages of mammoth eDNA would be unlikely to be so geographically uneven or to become geographically restricted over time.

Second, if mammoth DNA was continually ‘leaking’ into deposits, it would probably be detected in most (if not all) of the stratigraphic layers that formed after its DNA first found until the remains (whether preserved on the surface or exhumed from below) had disappeared altogether. Thus, mammoth DNA  would not be restricted to time-specific depositional layers within sites, but would instead be ‘smeared’ across successive layers. We do not see this either—there is no evidence of mammoth DNA being smeared throughout a section, either horizontally or vertically (Fig. 1b). Instead, the DNA of mammoths and other animals is usually restricted to specific strata and separated by layers where their DNA is absent, including fluvial sites that can harbour eDNA from geographically wider catchments and upstream DNA sources that may feed them^8^. In many cases, mammoth DNA is detected only in some—and not all—of the samples from the same stratum (Fig. 1b), indicating that it has not diffused through a horizontal layer.

Third, if mammoth DNA was an artefact of redeposition, the signal would probably be random with respect to changes in vegetation and climatic conditions. That is not the case. Our eDNA results were embedded in a comprehensive reconstruction of past Arctic ecosystems, which revealed continental and regional associations between mammoth eDNA and (1) eDNA of other animals, (2) the steppe-specific herbaceous plants, and (3) palaeo-climate panels reconstructed independently from different climate models (figures 2 and 4 in ref. ^3^). Our results show that the range of where mammoth eDNA has been found shrinks through the Holocene along with the shrinking of the steppe-tundra vegetation and the climatic and hydrogeological conditions to which the species was adapted to in the Pleistocene^11^, thereby supporting the geographically uneven and increasingly restricted pattern just noted. If lingering mammoth bones had leached older eDNA ubiquitously, we should not have seen spatiotemporal co-occurrences of mammoth, steppe vegetation, and the cold and dry Pleistocene-like climate conditions.

Finally, if redeposition of DNA in younger deposits was a problem, the eDNA of late-surviving mammoths ought to reflect the full range of clades present in mammoth populations in the late Pleistocene. They do not. Instead, we find a consistent decline of mammoth mitochondrial haplogroup diversity from the Pleistocene into the Holocene to the point where only Clade 1DE remained, both on isolated islands and on continental Siberia (figure 4 in ref. ^3^). It is highly unlikely that this reduction in genetic diversity was because individuals harbouring the same haplogroup were the only ones whose DNA was being released into younger sediments over time. This finding instead conforms to a pattern of a species’ decline towards extinction.

In sum, we find all evidence pointing to the validity of the eDNA identifications of late-surviving Arctic megafauna reported in our original study^3^. However, we acknowledge the possibility that unburied or exhumed animal fossils can contribute DNA to younger sediment layers, and this should always be considered (along the lines we described in ref. ^3^). This is particularly important in cases in which the animal species targeted were abundant and widely distributed on the landscape, for fine-resolution reconstructions, and for studies relying primarily on fluvial sediments as the eDNA source.

## Reporting summary

Further information on experimental design is available in the Nature Portfolio Reporting Summary linked to this Article.

## Online content

Any methods, additional references, Nature Portfolio reporting summaries, source data, extended data, supplementary information, acknowledgements, peer review information; details of author contributions and competing interests; and statements of data and code availability are available at 10.1038/s41586-022-05417-2.

### Supplementary information

Reporting Summary

## Data Availability

All data analysed in this study are included in this article or have been published previously.
